# Artificial Intelligence in Cervical Cancer Screening and Diagnosis

**DOI:** 10.3389/fonc.2022.851367

**Published:** 2022-03-11

**Authors:** Xin Hou, Guangyang Shen, Liqiang Zhou, Yinuo Li, Tian Wang, Xiangyi Ma

**Affiliations:** ^1^ Department of Obstetrics and Gynecology, Tongji Medical College, Tongji Hospital, Huazhong University of Science and Technology, Wuhan, China; ^2^ Cancer Centre and Center of Reproduction, Development and Aging, Faculty of Health Sciences, University of Macau, Macau, Macau SAR, China

**Keywords:** cervical cancer, cervical intraepithelial neoplasia (CIN), artificial intelligence, deep learning, cytology, colposcopy, early screening and diagnosis

## Abstract

Cervical cancer remains a leading cause of cancer death in women, seriously threatening their physical and mental health. It is an easily preventable cancer with early screening and diagnosis. Although technical advancements have significantly improved the early diagnosis of cervical cancer, accurate diagnosis remains difficult owing to various factors. In recent years, artificial intelligence (AI)-based medical diagnostic applications have been on the rise and have excellent applicability in the screening and diagnosis of cervical cancer. Their benefits include reduced time consumption, reduced need for professional and technical personnel, and no bias owing to subjective factors. We, thus, aimed to discuss how AI can be used in cervical cancer screening and diagnosis, particularly to improve the accuracy of early diagnosis. The application and challenges of using AI in the diagnosis and treatment of cervical cancer are also discussed.

## 1 Introduction

Cervical cancer is one of the most common malignancies in women, with 604,000 new cases and 342,000 deaths in 2020 (1). It is the only cancer that can be eliminated *via* primary prevention strategies comprising a fully effective 9-valent human papillomavirus (HPV) vaccine, early detection, and timely treatment (2).

Almost all cases of cervical cancer are caused by persistent infection of the cervical epithelium with one of the 15 genotypes of the carcinogenic HPV. The four major steps in the development of cervical cancer are as follows: infection of the metaplastic epithelium at the cervical transformation zone, persistent HPV infection, progression of persistently infected epithelium to cervical precancer, and invasion through the basement membrane of the epithelium (3). The HPV vaccines can protect girls and young woman against infection with the HPV virus. But HPV vaccine coverage rate is very low at present (even in some developed countries) (2, 4) and the beneficiaries are limited to young women aged <26 in terms of 9-valent HPV vaccines. According to American Cancer Society, vaccinated women are also recommended that be screened the same as unvaccinated women because it is impossible to avoid risk completely (5). Hence, routine screening for cervical cancer is still important to women. Approximately 30% of cervical intraepithelial neoplasia (CIN) grade 3 lesions develop into invasive cancers within 30 years. Slow progression offers many opportunities for the detection and treatment of these lesions (6). Screening and treatment of precancerous lesions in women is a cost-effective way to prevent cervical cancer (7). Ideally, screening strategies should be able to detect early lesions that may develop into cervical cancer, while avoiding the detection of transient HPV infections and benign abnormalities that can lead to overtreatment and other hazards associated with screening (5). With the continuous improvement of screening techniques, there has been an increase in the detection rate of cervical cancer and a decrease in the mortality rate; however, most deaths occur in low- and middle-income countries (8). Despite new developments in effective screening programs, many of these cannot be implemented or maintained because of weak health infrastructure (9, 10). Moreover, the accuracy of manual screening is not always 100% (11), resulting in some related lesions that cannot be timely diagnosed. Thus, developing a more accurate and economical cervical cancer screening method is the main challenge for the early diagnosis of cervical cancer.

In recent years, AI has been increasingly applied in the diagnosis of various diseases, such as the classification of skin tumors (12, 13), diagnosis and classification of retinal diseases (14), and imaging diagnosis of tumors (15), and has shown promising application value. AI can automatically recognize images, extract features, learn classification, and process data using complex algorithms. The application of AI in the early screening and diagnosis of cervical cancer is conducive to addressing limited human resources and improving diagnostic accuracy.

This article aimed to introduce recent AI technologies and demonstrate their utility and potential for the screening and early diagnosis of cervical cancer. This review also discusses the current challenges and proposes future research directions.

## 2 Methods for Cervical Cancer Screening and Diagnosis

**Figure 1:**
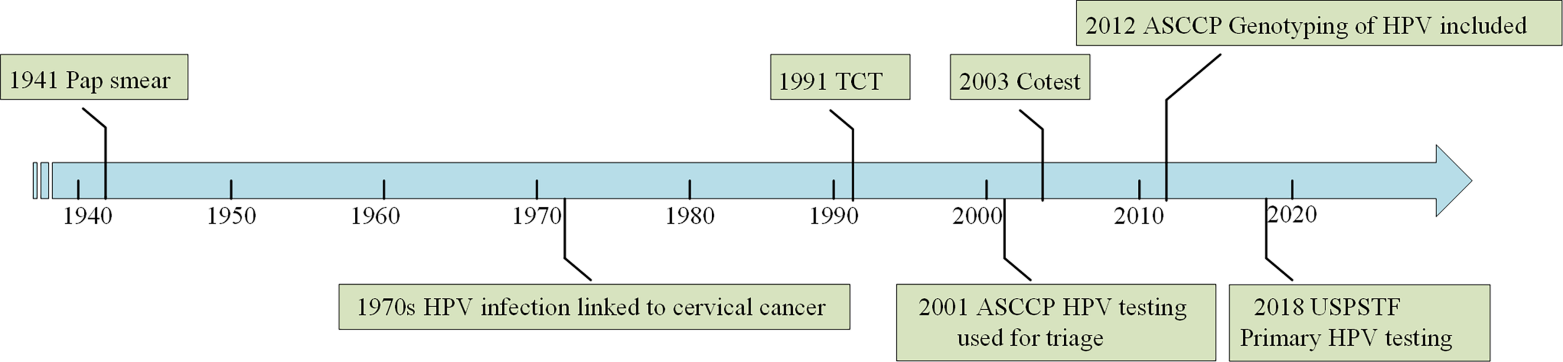
Evolution of cervical cancer screening methods. The figure shows major milestones in the evolution of cervical cancer screening. The main screening methods for cervical cancer are HPV testing and TCT (cytology) nowadays.

The latest World Health Organization guidelines recommend the following three screening methods for the early detection of cervical cancer: HPV testing, cytology (including traditional pap smear and liquid-based cytology smear), and visual inspection with acetic acid (VIA) (16). We focused on the first two methods because VIA is only used when the first two are not available. HPV testing and cytology uses brushed exfoliated cells from the cervix as test samples. HPV testing detects high-risk types of HPV infection in the cervix, whereas cytological examination uses a microscope to identify cells taken from the cervix for possible cervical cancer or precancerous lesions (17). **Figure 1** shows the evolution of cervical cancer screening methods. However, colposcopy-guided biopsy remains as the gold standard for cervical cancer diagnosis, followed by staging according to the clinical examination and imaging results.

### 2.1 Introduction to Cytology

A conventional Pap smear (CPS) is a manual screening procedure used to identify and classify exfoliated cervical cells under a microscope according to the color and characteristics of the nucleus and cytoplasm (18). Liquid-based cytology (LBC) can improve preparation techniques (19). The LBC specimen is better fixed in glass slides, easier to preserve and perform artificial removal, and has a more uniform sample distribution than CPS (20).

Cytology results are described according to the Bethesda system (TBS) (21). Cervical cells are grouped into specific categories according to their abnormal changes about nuclear size, degree of dyeing etc (22). Abnormal epithelial cells include atypical squamous cells and atypical glandular cells. The TBS nomenclature details are listed in **Table 1**.

**Table 1:** The Bethesda system.

Cell types	Classification
Normal	
Atypical squamous cells (ASC)	(1) atypical squamous cells of uncertain significance (ASC-US) (2) Highly squamous intraepithelial disease (ASC-H) cannot be ruled out; (3) Low-grade squamous intraepithelial lesions (LSILs, equivalent to CIN1); (4) Highly squamous intraepithelial lesions (HSILs), including CIN2 CIN3 and carcinoma in situ (CIS); (5) Squamous cell carcinoma (SCC)
Atypical glandular cells (AGC)	(1) Atypical glandular cells, not otherwise specified (AGC-NOS); (specify endocervical, endometrial, or not otherwise specified) (2) Atypical glandular cells, favor neoplastic; (specify endocervical or endometrial) (3) Endocervical adenocarcinoma in situ (AIS); (4) adenocarcinoma (specify endocervical, endometrial, extrauterine, or not otherwise specified).

### 2.2 Introduction to Colposcopy

Colposcopy is defined as the use of a specific instrument to magnify the fully exposed cervix by 5 to 40 times for a real-time visual assessment of the cervix, especially the transformation area, for the detection of CIN or squamous intraepithelial lesion (SIL) and invasive cancer (23). A colposcopy-guided biopsy of the suspected site is performed to determine whether further treatment, such as conization or cryotherapy, is needed, which is important in patients with high-grade CIN or more severe disease (24).

### 2.3 Procedures for Early Screening and Diagnosis of Cervical Cancer

**Figure 2:**
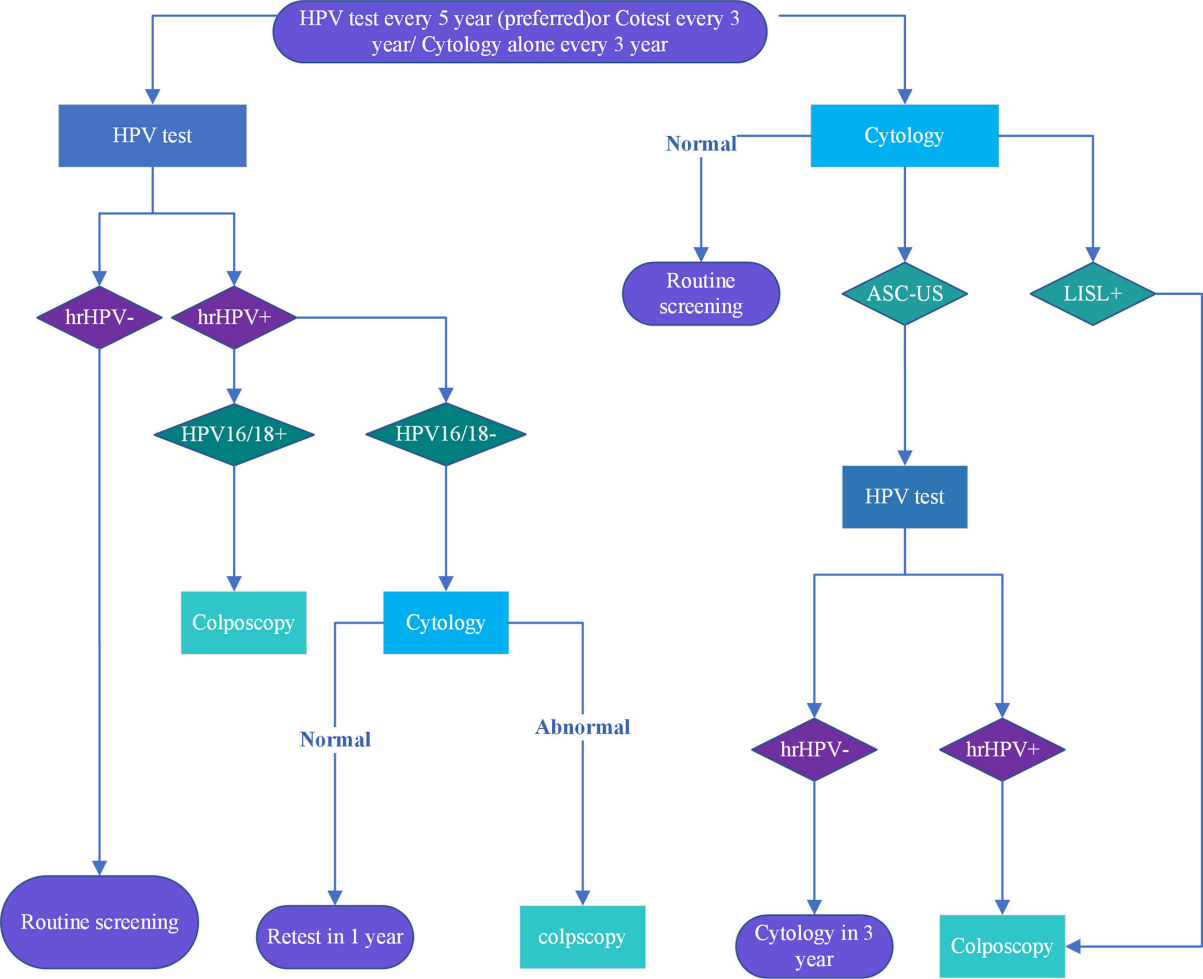
Cervical cancer screening procedures are recommended for women aged 25 to 65. The American Cancer Society recommends screening starting at age 25 Colposcopy is recommended for HPV16/18 +, ASC-US and high risk HPV+ cytology with cytological results above ASC-H. Re-screening is recommended after 1 year for other abnormalities, and after 3 years for normal ones.

According to the latest recommendations of the American Cancer Society on cervical cancer screening, women with a cervix aged ≥25 years are recommended to undergo cervical cancer screening. For women between the ages of 25 and 65, primary HPV test should be performed every five years (5). If a primary HPV test is not available, co-testing (HPV testing in combination with cytology) or cytology evaluation can be performed every three years. Colposcopy or recommended screening methods can be performed based on the results (25), which are shown in the **Figure 2**.

Referral colposcopy further determines the presence of CIN and identifies or excludes invasive carcinoma (4). Patients with a high suspicion of invasive cancer should undergo colposcopy-guided biopsy, the gold standard for the diagnosis of cervical cancer and plays a key role in the early detection of cervical cancer (26).

## 3 Applications of AI in Early Screening of Cervical Cancer

### 3.1 HPV Typing and Detection

Continuous high-risk HPV infection can lead to cervical cancer (27). HPV testing can detect HPV infection and help screen high-risk populations. Genotyping of HPV will make it easier to assess the risk of women with positive cervical smear results and HPV DNA-positive results (28), thus making it more conducive for cervical cancer screening and management. AI learning technology uses research related to HPV testing to improve accuracy and diversify the use of HPV testing in cervical cancer screening. These studies are summarized in **Table 2**.

**Table 2:** Application of AI in HPV testing.

Reference	Year	Aim of study	Number of subjects	Methods	Results
Wong et al. (29)	2019	Identifying high-grade lesions and in triaging equivocal smears	605 cervical cytology samples	Decision tree, random forest SVM-linear SVM-nonlinear	Specificity: 94.32% Specificity: 90.91% Specificity: 90.91% Specificity: 90.91%
Pathania D et al. (30)	2019	Point-of-care HPV screening	Training sets: 13000 images Validation: 35 cervical specimens	CNN	Sensitivity: down to a single cell specificity: 100%
Tian R et al. (31)	2019	Predicting cervical lesion grades	10 HPV+ cases 10 CIN1 cases 14 CIN2+cases	Random forest unsupervised clustering	Accuracy 0.814

HPV, human papillomavirus; CIN, cervical intraepithelial neoplasia; SVM, support vector machine; CNN, convolutional neural network.

Different types of HPV are associated with different types of lesions. For example, cervical adenocarcinomas are usually associated with HPV 18 type and tend to shed fewer cells; thus, they are difficult to detect by cytology (32). Having high-risk HPV types (e.g., 16, 18, and 31) contributes to a greater risk of developing cervical malignancies (11). Therefore, distinguishing among the specific types of HPV will make it easier to classify and manage HPV-infected women. Based on the additional genotyping information provided by Onclarity, Wong et al. derived a decision system with 94.32% specificity of the best classifier. The system highlighted the patients who are at high risk of developing CIN2/3+, demonstrated that some infections involving multiple HPV types carry additional risks, and identified the most important gene combinations (29). Pathania proposed the HPV AI surveillance, which uses a deep learning (DL) algorithm combined with digital micro-holography, and reported excellent sensitivity and specificity (100% coincidence) in detecting HPV 16 DNA and HPV 18 DNA in cell lines (30).

However, more studies are underway. The high sensitivity of HPV testing results in an increased rate of colposcopy referrals, which may lead to more potentially harmful treatments (33). Tian et al. analyzed HPV integration status, somatic mutation, and copy number variation through capture-based next-generation sequencing and obtained enriched biomarkers of CIN 2. They then used a machine learning algorithm (random forest) to build a risk stratification model for cervical precursor lesions, which successfully predicted CIN2+ with an average accuracy probability score of 0.814 (31). This method effectively stratified the risk of cervical lesions and provided valuable integrated triage strategies.

At present, HPV typing mostly depends on test kits; however, these have some disadvantages, such as false-negative rate and high cost. AI has shown great potential and application in the detection of HPV types and associated molecular markers that can aid in the diagnosis of cervical lesions.

### 3.2 Screening of Cervical Cytology

Cytology-based cervical cancer prevention programs have reduced the incidence of cervical cancer in many Western countries (34). Cytology screening for high-grade cervical precancerous lesions is highly specific but less sensitive (50–70%) (11) and requires careful microscopy observation by well-trained cytologists. Each process is cumbersome, labor-intensive, and error-prone (35). In addition, cytological reproducibility is low, resulting in low accuracy (36). Furthermore, changing the observer yields inconsistent and subjective results (37). So the researchers hope to develop automatic image analysis methods to relieve these pressures.

The first commercial automatic screening system was PAPNET (38) in 1992. The system was approved as a method of re-screening for slides that were judged negative by cytologists. In 2004, FAD approved the Thinprep imaging system as a commercial screening product. The system can select the 22 most concerned fields of view (FOV) according to the proprietary algorithm, and if abnormal cells are found, cytotechnologists need to manually screen the entire slide (39). The system improves the sensitivity and efficiency of screening. Later, the Focal point GS imaging system emerged in 2008. It identified 10 FOV of cervical cells most likely to be abnormal and stratified the risk to improve the efficiency (40). However, some reviews indicate that the cost-effectiveness of these automation systems is limited and is not suitable for use in low-and medium-developing countries (41). And its research technology still has weaknesses (42) and still depends on the final manual screening process. Therefore, some researchers continue to optimize the application of artificial intelligence technology in cervical cytology.

#### 3.2.1 Segmentation of Cervical Cells

A typical automatic smear analysis system comprises the following five stages: image acquisition, preprocessing, segmentation, feature extraction, and classification (43). AI technology is applied in the segmentation and classification stages for the automatic analysis of a smear, which is helpful to improve screening efficiency.

**Figure 3:**
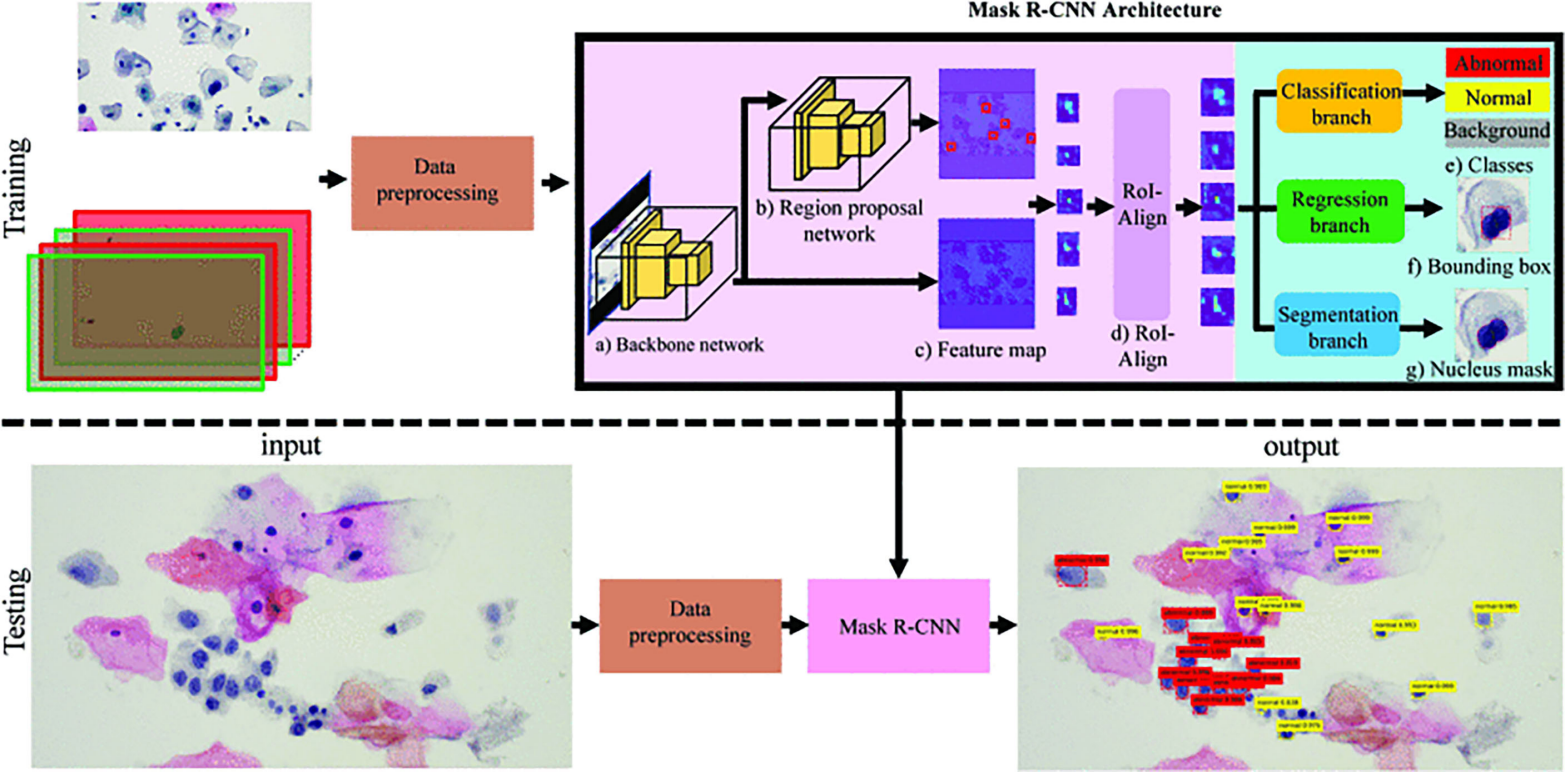
A example segmentation model based on Mask-RCNN architecture. Reproduced with the permission of ref. (51), copyright@IEEE, 2019. In training phase, input was pap smear slide image and nucleus ground truth mask with class label was preprocessed and then trained in Mask R-CNN. In testing phase, pap smear slide image was preprocessed. Mask RCNN was used to specify bounding box, nucleus mask, and class of each cell.

The first step in cytological diagnosis is the accurate identification of cells and their respective structural components. As the diagnostic criteria for cervical cytology are mainly based on nuclear and cytoplasm abnormalities, accurate segmentation is a prerequisite for screening solutions (44–46). Studies on the application of AI in cell segmentation have been carried out continuously and have shown good results in the segmentation of hepatoma cells (47), human metaphase II oocytes (43), and pluripotent stem cells (48). It has also been introduced for the automatic segmentation of cervical cells, and good results have been reported (49). For example, Chankong et al. used fuzzy c-means clustering technology to segment single-cell images into the nucleus, cytoplasm, and background to realize whole-cell segmentation (41). Some researchers extracted adaptive shape from cytoplasmic contour fragments and shape statistics to segment the overlapped cytoplasm of cells in cervical smear images using supervised learning. Experimental results show that this method is efficient and always superior to the most advanced methods (50). Segmentation model on images from pap smear slide also was explored which has been achieved through using nucleus localization to classify normal and abnormal cells, combined with single cell classification algorithm. The accuracy and sensitivity are 91.7% respectively and the model consists of two stages as shown in Mask-RCNN architecture part in **Figure 3** (51). Review of relevant literature results are provided in **Table 3**.

**Table 3:** Application of AI in cervical cell segmentation.

Reference	Year	Number of subjects	Methods	Datasets	Results
Wang et al. (44)	2014	362 cervical cell images (3722 cells)	Mean-Shift clustering algorithm	Private	Sensitivity: 94.25% Specificity: 93.45%
Song et al. (50)	2019	8 cervical cell images 22 cervical images	CNN	ISBI2015 Private	DSC: 0.84 DSC: 0.83
Zhao et al. (45)	2016	917 single-cell images	Superpixel-based Markov random field	Herlev The real-word Datasets	Herlev ZSI of nuclei: 0.93 ZSI of cytoplasm: 0.82
Gautam et al. (46)	2018	917 single-cell images	Patch-based CNN	Herlev	DSC: 0.90 Precision: 89%

CNN, convolutional neural network; DSC, dice similarity coefficient; ZSI, zijdenbos similarity index.

AI makes cervical cell automatic segmentation true, accurate, and unified. Thus, time-intensive manual segmentation process and subjective shortcomings can be overcome to realize accurate abnormal cell classification.

#### 3.2.2 Classification of Cervical Cells

Accurate classification of cervical cells in smears is a crucial step in cervical cancer screening. The low accuracy of manual classification and the high requirement for professional degree of the observer limit the application of cytology (52), particularly in areas where trained cytopathologists are scarce. The use of AI has addressed these limitations (**Table 4**).

**Table 4:** Application of AI in cervical cell classification.

Reference	Year	Methods	Datasets (Num. of images)	Classes	Results
Chankong et al. (41)	2014	Bayesian classifier KNN ANN	ERUDIT (552)	4-class	Accuracy 96.20%
				2-class	Accuracy 97.83%
			Herlev (917)	7-class	Accuracy 93.78%
				2-class	Accuracy 99.27%
			LCH (300)	4-class	Accuracy 95.00%
				2-class	Accuracy 97.00%
Borakden et al. (53)	2017	Ensemble classifier: LSSVM MLP RF	Cell level (1610)	2-class	Accuracy 99.07%
					Specificity 98.90%
			Smear level (1320)	3-class	Accuracy 98.11%
					Specificity 99.35%
			Hervel (917)	2-class	Accuracy 96.51%
					Specificity 89.67%
Zhang et al. (54)	2017	CNN; Transfer learning	Herlev (917)	7-class	Accuracy 98.30%
					Specificity 98.30%
			HEMLBC (2370)	2-class	Accuracy 98.60%
					Specificity 99.00%
					sensitivity 98.30%
Hussain et al. (52)	2020	CNN; Transfer learning	LBC (own) (1670), Conventional(own) (1320)	4-class	Accuracy 98.90%
					Sensitivity 79.80%
					Specificity 97.90%
Shi J et al. (55)	2020	CGN	SIPAKMeD (4049)	5-class	Accuracy 98.37%
					Sensitivity 99.80%
			MOTIC (25378)	7-class	Accuracy 94.93%
					Sensitivity 92.98%
Rahaman et al. (56)	2021	HDFF	Herlev (917)	2-class	Accuracy 98.32%
				7-class	Accuracy 90.32%
			SIPAKMeD (4049)	2-class	Accuracy 90.32%
				5-class	Accuracy 99.14%

KNN, K- Nearest Neighbor; ANN, Artificial Neural Network; LSSVM, Least Squares Support Vector Machine.

CNN, convolutional neural network; CGN, graph convolution network; HDFF, hybrid deep feature fusion techniques.

In the past few decades, many classification methods have been proposed, most of which are based on segmentation or texture feature extraction. Chankong et al. segmented the cervical single-cell image into the nucleus, cytoplasm, and background, and obtained the morphological features to realize automatic multi-label classification. The results showed an accuracy rate of more than 93% (44). Mariarputham et al. extracted seven groups of texture features of cervical cells for classification. The support vector machine (SVM) classifier had the highest accuracy and the best performance (57). Three classifiers, i.e., least square support vector machine (LSSVM), multilayer perceptron (MLP) and random forest (RF), were used in the integrated classifier designed by Kden et al. The accuracy of these classifiers was 98.11% at the smear level and 99.01% at the cell level (53). Reducing the time of manual observation eliminates observer bias and improves efficiency.

Classification that does not rely on an accurate segmentation algorithm has also been proposed and accepted by an increasing number of scholars. Zhang et al. applied DL and transfer learning (58) to cervical cell classification for the first time. Automatic extraction of embedded deep-level features in cell images for classification is a superior method compared with previous algorithms in terms of classification accuracy (98.3%), AUC (0.99), and specificity (98.3%) (54). Six different convolutional neural networks (CNNs) were used for the first time for the diagnosis of cervical precancerous lesions. The accuracy, sensitivity, and specificity of the integrated classifier were 0.989, 0.978, and 0.979, respectively (43). Shi et al. proposed a method of cervical cell classification based on a graph convolution network to explore the potential relationship between cervical cell images and improvement of classification performance. Its accuracy (98.37%), sensitivity (99.80%), specificity (99.60%), and *F*-measure (99.80%) were all better than those of the existing method (55). In addition, hybrid deep feature fusion techniques were proposed, with high accuracy in the SIPAKMeD dataset (56).

#### 3.2.3 AI Improves the Screening accuracy of Cervical Intraepithelial Lesions

After the establishment of a good automated cytological detection model based on the AI method, some studies have found that cytological examination assisted by AI can classify cervical cells to guide triage and improved the detection rate of CIN compared with that of standard pathological biopsy results (32, 59) (**Table 5**).

**Table 5:** Application of AI in cytology to detect CIN.

Reference	Year	N	Methods	Databases	Results
Yu et al. (32)	2018	1839	Risk score algorithm	Cytological image HPV testing	CIN2+ AUC 0.710CIN3+ AUC 0.740
Bao et al. (60)	2020	703103	DL	Cytological image	CIN1+ Sensitivity 88.9%Specificity 95.8%CIN2+ Sensitivity 90.10%Specificity 94.80%CIN3+Sensitivity 90.90%Specificity 94.40%
Bao et al. (61)	2020	2145	ResNet	Cytological image	CIN2+ AUC 0.762CIN3+ AUC 0.755
Wang et al. (62)	2020	143	DL	whole slide images (WSIs)	precision 93.00%recall 90.00%, F-measure 88.00%
Holmström O et al. (59)	2021	740	DL	Cytological image	HSIL+ AUC 0.970Sensitivity 85.7%Specificity 98.5%
Zhu et al. (63)	2021	980	AIATBS	Cytological imageBiopsy diagnosis results	Sensitivity 94.74%

DL, deep learning; CNN, convolutional neural network; AIATBS, artificial intelligence-assisted TBS; CIN, cervical intraepithelial neoplasia; AUC, area under the curve; HSIL, high squamous intraepithelial lesion.

A prospective cohort study of 700,000 women in a population-based cervical cancer screening program using a validated AI-assisted cytological diagnostic system was conducted by Bao et al. They reported a total coincidence rate of 94.7% and a 5.8% increase in sensitivity (3.0–8.6%) compared to manual reading (60). Another observational study was conducted to evaluate the ability of AI-assisted cytology to histologically detect CIN or cancer. Detection rates for CIN 2 and CIN 3+ of 92.6% and 96.1%, respectively, were obtained. These were significantly higher than those of manual reading (61). Furthermore, Wang et al. established a DL-based cervical disease diagnosis system that detects high squamous intraepithelial lesions (HSILs) or higher, and an accuracy of 0.93 was achieved (62). Zhu et al. developed an AI-assisted TBS (AIATBS) (21) diagnostic system, which showed higher sensitivity than the diagnosis by senior cytologists. The sensitivity of the AIATBS in detecting CIN was 94.74% in a clinical prospective validation (63).

The evidence above overall indicates that AI has been widely used for HPV testing and cytology and has achieved a good detection rate and accuracy. More studies and applications are underway in this regard. For example, Tang et al. developed an AI microscope with an augmented reality (AR) display for cervical cytology screening. They reported that it significantly improved detection sensitivity for low squamous intraepithelial lesion (LSIL) and HSIL and consistency in multiple classifications and atypical squamous cells of uncertain significance recognition. In addition to diagnostic applications, training novice cytopathologists could be another potential application for AI microscopes (64).

## 4 Applications of AI in Cervical Cancer Diagnosis

**Figure 4:**
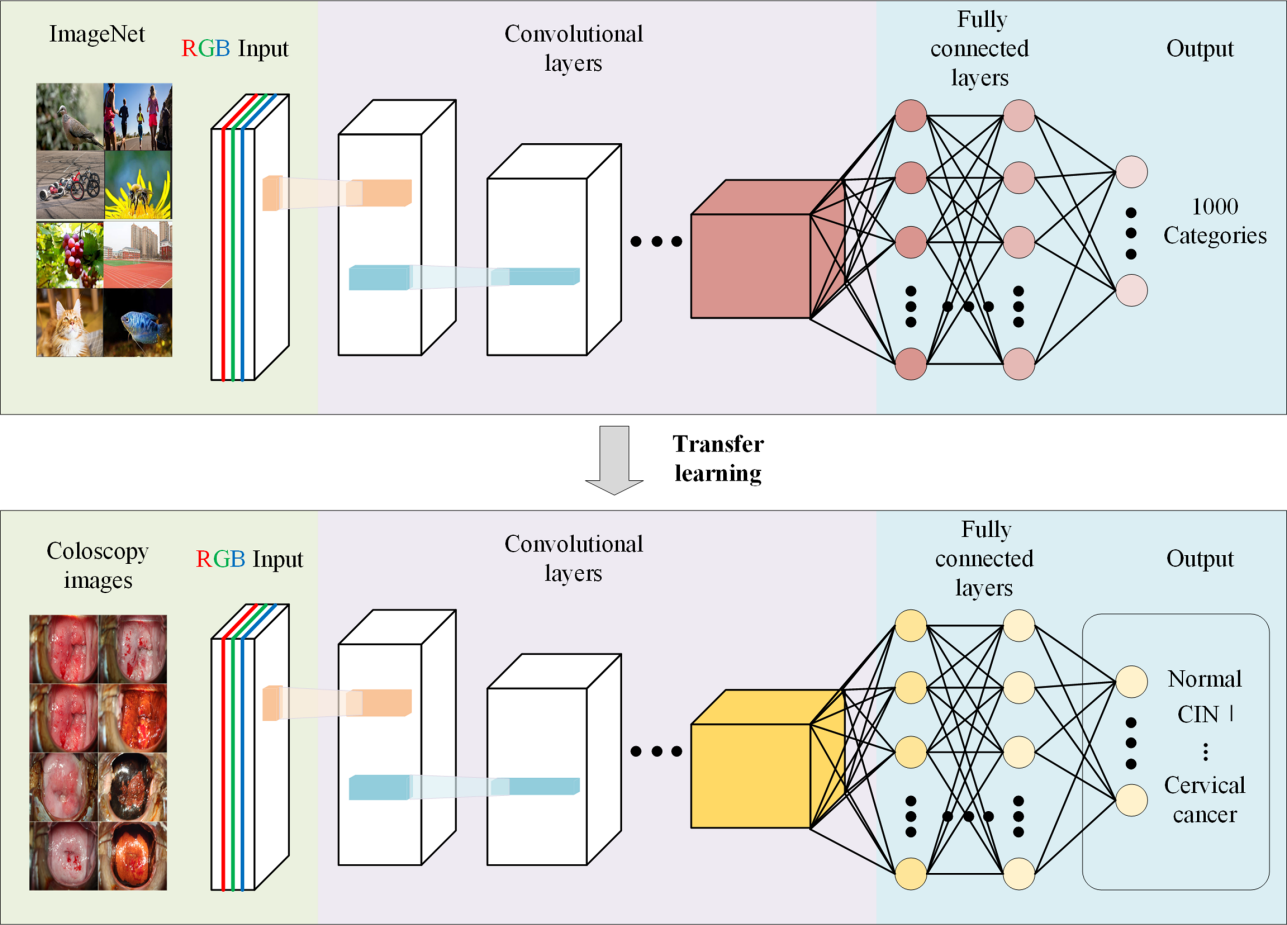
Schematic representation of application of Convolutional Neural Network in colposcopy images. Schematic depicting that a CNN pre-trained on other large-scale image datasets can be adapted to significantly increase the accuracy and shorten the training duration of a network trained on a novel dataset of colposcopy images.

Cervical cancer and precancerous lesions are diagnosed by colposcopy-guided biopsy and staged according to the Federation International of Gynecology and Obstetrics (FIGO) staging standard (65). In 2018, the FIGO allowed the use of imaging and pathologic findings (if any) in staging (66). AI technology has been used in colposcopy and magnetic resonance imaging (MRI) to assist in the diagnosis and staging of cervical cancer and has shown satisfactory results. **Figure 4** illustrates the workflow of the CNN model and transfer learning used in colposcopy image classification.

### 4.1 Coloscopy

At present, the consistency between colposcopy and pathology is poor, which may lead to misdiagnosis and missed diagnosis. Colposcopy performed by an unskilled clinician could result in potential harm (including bleeding, infection, vaginal discharge, pain, or discomfort) and therefore, requires adequate training and experience to achieve proficiency and ensure maintenance of operating procedures. However, the long training period of professional colposcopy doctors and the lack of qualified personnel create challenges for the use of colposcopy in the diagnosis of cervical cancer (67).

#### 4.1.1 AI Boosts Image Classification Performance

Recently, DL has been widely used in medical imaging (15). The application of DL technology in the classification of colposcopy is helpful in solving the bottleneck of traditional colposcopy and improving its diagnostic performance (**Table 6**).

**Table 6:** Application of AI in colposcopy.

Reference	Year	Aim of the study	Number of subjects	Methods	Images	Results
Kim E et al. (68)	2013	Detection of CIN2+	2000images	SVM	Cervicography	Sensitivity 75.00%Specificity 75.00%
Song et al. (69)	2015	Detection of CIN2+	7669patients	MCNN	Cervicography	Accuracy 80.00%Sensitivity 83.21%Specificity 94.79%
Hu et al. (70)	2019	Detection of CIN2+	9406patients	Faster-CNN	Cervicography	AUC 0.91
Chao et al. (71)	2020	Detection lesions need to biopsy and classification	791 patients	CNN	Optical colposcopy image	Sensitivity 85.20%Specificity 88.20%AUC 0.947
Asiedu et al. (72)	2019	Classification of cervical lesions	134 patients	SVM	Digital colposcopy images	Accuracy 80.00%Sensitivity 81.30%Specificity 78.60%
Yuan et al. (26)	2020	Classification of cervical lesions	22330images	CNN	Digital colposcopy images	Sensitivity 85.38%Specificity 82.62%
Miyagi et al. (73)	2019	Classification of cervical lesions	253patients	CNN	Traditional colposcopy images	Accuracy 83.30%Sensitivity 95.60%
Miyagi et al. (23)	2019	Classification of cervical lesions	310images	CNN	Traditional colposcopy images	Accuracy 82.30%Sensitivity 80.00%Specificity 88.20%
Xue et al. (74)	2020	Classification of cervical lesions	19435patients	CAIADS	Digital colposcopy images	LSIL Sensitivity 90.50%Specificity 51.80%HSIL Sensitivity 71.90%Specificity 93.90%
Yue et al. (75)	2020	Classification of cervical lesions	4753images	CNN,	cervigram images	Accuracy 96.13%Sensitivity 95.09%Specificity 98.22%AUC 0.94
Venkatesan et al. (76)	2021	Classification of cervical lesions	5679images	CNN	colposcopy photographs	Accuracy 83.30%Sensitivity 95.60%
Peng et al. (77)	2021	Classification of cervical lesions	300images	VGG16	colposcopy images	Accuracy 86.30%Sensitivity 84.10%Specificity 89.80%

CAIADS, Colposcopic Artificial Intelligence Auxiliary Diagnostic System.

Miyagi et al. developed and trained a CNN AI classifier for the LISIL/HSIL classification of colposcopy images. The accuracy, sensitivity, and specificity of the AI classifier and oncologist in diagnosing HSIL were 0.823 and 0.797, 0.800 and 0.831, and 0.882 and 0.773, respectively (24). Later, a classifier based on DL was developed, which uses HPV types and cervical SIL images to classify HSIL/LSIL. The accuracy of the classifier was 0.941 (73). Xue et al. developed an AI method (CAIADS) to grade the colposcopy impression and guide the biopsy. The consistency of CAIADS-graded colposcopy impression and pathological results (82.2%) was higher than that traditional colposcopy (65.9%) (74). The ResNet model based on DL was established by Yuan et al. Its sensitivity and specificity based on DL were 85.38% and 82.62%, respectively. The model helps in colposcopy diagnosis and guides biopsy (26). The C-RCNN algorithm was proposed by Yue et al. to classify cervical lesions, and the time and spatial features were extracted using long-term and short-term memory network. Models with better specificity (98.22%), sensitivity (95.09%), accuracy (96.13%), and area under the curve (0.94%) were obtained (75).

#### 4.1.2 AI Helps Detect High-Grade Cervical Lesions and Guides Biopsy

Clinically, one of the most important goals of cervical cancer screening is to distinguish between normal/CIN 1 and CIN 2/3+. If the lesion is classified as CIN 2/3+, treatment is required. In contrast, mild dysplasia in CIN 1 is usually cleared following approximately a year of immune response and can therefore be observed or treated more conservatively. Kim et al. developed a data-driven computer interpretation algorithm for cervical images based on color and texture. They obtained a sensitivity of 74% and a specificity of 90% in differentiating high-grade cervical lesions (CIN 3+) from low-grade lesions and normal tissues (68). Hu et al. conducted a longitudinal cohort study on 9,406 women for 7 years. The cervical images obtained were used to validate the model based on the fast R-CNN method. The AUC of the model for diagnosing CIN 2+ was 0.91, which exceeded the interpretation of the same image by the colposcope evaluator and was superior to those of traditional Pap smears and alternative types of cytology (70). Cho et al. developed a binary decision model to determine the need to biopsy for a cervical lesion. The Need-To-Biopsy was defined as ‘not being normal’, referring to CIN+ and LSIL+. The performance of the best RESNET-152 model showed an average AUC of 0.947, a sensitivity of 85.2%, and a specificity of 88.2% (71); thus, the model helps an inexperienced clinician judge whether to perform a cervical biopsy or refer the patient to a specialist.

The powerful image analysis ability of AI has solved the problem of diagnosing cervical cancer using a large number of colposcopy images. With the assistance of AI technology, the accuracy of detecting lesions and performing biopsy under colposcopy becomes relatively high, thus reducing the misdiagnosis rate of colposcopy (69, 72, 76, 77).

### 4.2 Pelvic MRI

MRI has proven to be highly accurate in the preoperative staging of cervical cancer (78, 79). Therefore, MRI is the first choice for local staging, evaluation of treatment response, detection of tumor recurrence, and follow-up of patients with cervical cancer (80). The primary objective of MRI is to determine the presence of peritumoral infiltration and lymph node metastasis (LNM) (81) (**Table 7**).

**Table 7:** Application of AI in MRI to diagnosis cervical cancer.

Reference	Year	Aim of study	Number of cases	Methods	Results
Lin et al. (82)	2020	Cervical Cancer MRI Image segmentation and location	169 patients (training set 144; validation set 25)	DL Radiomics	A dice coefficient: 0.82; Sensitivity: 0.89, PPV:0.92
Wang et al. (83)	2020	Segmentation: Prediction of parametrial invasion	137 patients (training set 91; validation set 46)	Radiomics	Training set AUC T2WI: 0.797 T2WI and DWI0.780 (95% CI)Validation set T2WI 0.946 (95% CI) T2WI and DWI 0.921 (95% CI)
Peng et al. (84)	2019	Enhancing Cervical Cancer MRI Image Segmentation	Not mention	Wireless network; DL	AUC 0.980
Yu et al. (85)	2019	Assisting diagnosis of lymph node metastasis	153 patients (training set 102; validation set 51)	Radiomics	Training set AUC: 0.870Validation set AUC 0.864
Wu et al. (86)	2019	Assisting diagnosis of lymph node metastasis	189 patients (training set 126; validation set 63)	Radiomics	Training set AUC 0.895 Sensitivity 94.3%Validation set AUC 0.847 Sensitivity 100%
Wang et al. (87)	2019	Assisting diagnosis of lymph node metastasis	96 patients (training set 96; validation set 96)	RadiomicsSVM	Training set C-index 0.893(P=4.311*10^-5^)Validation set C-index 0.922(P=3.412*10^-2^)
Xiao et al. (88)	2020	Assisting diagnosis of lymph node metastasis	233 patients (training set 155; validation set 78)	Radiomics	Training set C-index 0.856 (95% CI)Validation set C-index 0.883 (95% CI)
Wu et al. (89)	2020	Assisting diagnosis of lymph node metastasis	479 patients (training set 338; validation set 141)	DL	AUC 0.933 (95% CI)

#### 4.2.1 Segmentation of Cervical Cancer Lesions

MRI has a higher soft tissue resolution than CT. It can determine tumor size and adjacent pelvic structures and assess periuterine invasion and uterine and vaginal involvement (68). Lin et al. developed a U-Net CNN to accurately locate and segment cervical carcinoma in diffuse-weighted imaging (DWI). They reported the highest learning efficiency during image training, with a dice coefficient of 0.8, sensitivity of 0.89, and a positive predictive value of 0.92 (82). Liang et al. established a computational model of a DL algorithm based on wireless network, which can be used to segment cervical cancer MRI images with a 98% accuracy, which is evidently better than that of traditional depth-learning algorithms (<90%) (84). AI is more accurate, objective, and faster than manual segmentation. Wang et al. established a non-invasive radiologic model based on T2-weighted imaging (T2WI) and DWI, divided MRI images, and extracted features to predict periuterine invasion. The AUCs of T2WI and T2WI combined with DWI in the validation cohort were 0.780 and 0.921, respectively (83).

#### 4.2.2 Diagnosis of Cervical Cancer LNM

AI also contributes to the early diagnosis of cervical cancer LNM. Although the accuracy of computed tomography and MRI in assessing lymph node involvement only ranged from 83% to 85%, their specificity was very high, ranging from 66% to 93% (74). In 2018, the cervical cancer staging system was revised to include lymph node status as a staging criterion for the first time. Cervical cancer with lymph node involvement on imaging or pathology was classified as stage IIIC (66). Wu et al. developed a DL model using preoperative MRI to predict LNM in patients with cervical cancer. The AUC using both intratumoral and peritumoral DL models in T1WI was 0.844, whereas that of the hybrid model, which combines the tumor image information from DL mining with the lymph node status reported by MRI, was 0.933 (89), thus improving the detection rate of LNM.

Over the past decade, radiology has evolved to bridge the gap between imaging and precision medicine. Radiology uses complex image analysis tools combined with statistical analysis to extract rich information hidden in medical images (90). Wu et al. used MRI radiomic analysis to improve the diagnostic level of LNM in patients with cervical cancer. The combination of T2WI and decision tree of lymph node status had the best diagnostic effect; in the training and validation cohorts, the AUC and sensitivity were 0.895% and 94.3% and 0.847% and 100%, respectively (89). Wang et al. showed that radiographic images based on T2WI and DWI showed a good predictive power for pelvic LNM in early cervical cancer (83). More values are listed in **Table 7** (85–89).

## 5 Limitations and Future Directions

AI performs well in both computing and image analyses. These characteristics make it a rising star in the field of medical research, helping clinicians in decision-making, reducing the workload of doctors, and reducing the rate of misdiagnosis. Overall, AI can improve the specificity and accuracy of screening and diagnostic programs, overcome time constraints and limited professional and technical personnel, and avoid bias caused by subjective factors, which will enable cervical cancer screening to be implemented in resource-poor areas, thus markedly reducing the incidence of cervical cancer.

However, the application of AI involves some challenges. First is data; machine learning (ML) algorithms sometimes lack data in that they typically require millions of observations to achieve acceptable performance levels (86). However, current clinical data may be scarce, lack markers, and have uncertain quality. The management of medical data is another major obstacle for the development of automated clinical solutions (15). The establishment of not only multiple but also standardized and large databases is a future concern. Data security issues and overfitting should also be considered as they can give rise to exaggerated results that can lead to overdiagnosis (90). Second, models established using AI have not been applied and popularized in clinical practice; therefore, a series of prospective clinical studies are urgently needed to verify these results. Third, AI cannot replace clinicians as it is only an auxiliary diagnostic approach. AI may also cause system paralysis, requiring technical maintenance skills. Furthermore, maintenance systems need to be trained and established.

AI is promising in cervical cancer screening, especially its application in cervical cytology is relatively mature. But the segmentation technology is still faced with many challenges which is important for automatic classification. Such as the segmentation of overlapping nuclei, the processing of non-target cells, and fragments and the quality control of slide dyeing differences are still problem that needs to be optimized. We also mentioned that some classification methods do not rely on segmentation techniques in the previous. It will avoid many pre-processing steps and may be a direction for future development. In addition to the application of early screening and diagnosis mentioned in this paper, AI can be applied to the treatment, prognosis prediction, and prevention of cervical cancer. In the future, more research on treatment and prediction is needed for better treatment decision-making. This will facilitate cervical cancer eradication programs worldwide. Furthermore, as the incidence of cervical adenocarcinoma and other rare pathological types increases, AI should be applied for the early diagnosis of such diseases in the future. AI can also be used for noninvasive differentiation of cervical cancer from other diseases. Further development of AI technologies will greatly enhance the prediction of cervical cancer, maximize the improvements in cervical cancer screening and diagnosis, optimize staging systems, and improve patient prognosis.

## Author Contributions

XH and YL contributed to the collection of relevant literature. GS and YL contributed to literature analysis. XH reorganized the literature and wrote the original draft of the manuscript. LZ and TW provided considerable help in the revision of the manuscript. XM was responsible for the design of the review and revision of the manuscript. All authors contributed to the article and approved the submitted version.

## Funding

This work was supported by the National Key Research & Development Program of China (grant number 2021YFC2701402).

## Conflict of Interest

The authors declare that the research was conducted in the absence of any commercial or financial relationships that could be construed as a potential conflict of interest.

## Publisher’s Note

All claims expressed in this article are solely those of the authors and do not necessarily represent those of their affiliated organizations, or those of the publisher, the editors and the reviewers. Any product that may be evaluated in this article, or claim that may be made by its manufacturer, is not guaranteed or endorsed by the publisher.
